# Prevalence, predictors, and clinical relevance of α‐gal sensitization in patients with chronic urticaria

**DOI:** 10.1002/clt2.12199

**Published:** 2022-10-25

**Authors:** Helena Swee‐Lin Trige Pedersen, Jennifer Astrup Sørensen, Flemming Madsen, Allan Linneberg, Katja Biering Leth‐Møller, Christian Vestergaard, Simon Francis Thomsen

**Affiliations:** ^1^ Department of Dermato‐Venereology and Wound Healing Centre Copenhagen University Hospital Bispebjerg Copenhagen Denmark; ^2^ Allergy and Lung Clinic, Madsen & Ringbæk Elsinore Denmark; ^3^ Center for Clinical Research and Prevention Copenhagen University Hospital – Bispebjerg and Frederiksberg Copenhagen Denmark; ^4^ Department of Clinical Medicine Faculty of Health and Medical Sciences University of Copenhagen Copenhagen Denmark; ^5^ Department of Dermatology Aarhus University Hospital Aarhus Denmark; ^6^ Department of Biomedical Sciences University of Copenhagen Copenhagen Denmark

**Keywords:** α‐gal, alpha‐gal‐syndrome, galactose‐α‐1,3‐galactose, IgE, red meat allergy, urticaria

## Abstract

**Background:**

Little is known about α‐gal (galactose‐α‐1,3‐galactose) sensitization in patients with chronic urticaria (CU). The aim of this study was to examine the prevalence, predictors and clinical relevance of α‐gal sensitization in patients with CU.

**Methods:**

Two consecutive cohorts of newly referred patients with CU from a primary care allergology practice and a tertiary hospital dermatology department, plus a control group with allergic disease, but not CU, from the allergology practice, were interviewed and screened for α‐gal sensitization (serum specific‐IgE ≥0.35 KU/L).

**Results:**

Of 733 patients included, 21 (5.6%) and 11 (3.9%) of CU patients from private practice and hospital, respectively, were α‐gal sensitized. In total, 8 patients (38.1% of sensitized patients, and 2.1% of all CU patients) from private practice, and 2 patients (18.2% of sensitized patients, and 0.7% of all CU patients) from hospital, had clinically relevant α‐gal allergy. In private practice, male sex (47.6 vs. 24.7%), *p* = 0.020, obesity (33.3 vs. 23.6%), *p* = 0.302, and frequency of angioedema (61.9 vs. 51.4%), *p* = 0.350; and in hospital, male sex (72.7 vs. 27.9%), *p* = 0.003, and high total immunoglobulin E (median 168 vs. 70.5 KU/L), *p* = 0.022 were associated with α‐gal sensitization.

**Conclusion:**

α‐gal sensitization is observed in a small fraction of CU patients with only few patients experiencing clinically relevant sensitization. Certain patients, particularly from primary care, may constitute a relevant population for aimed testing.

To the Editor,

1

Chronic urticaria (CU) is a severely itching skin disease characterized by wheals, angioedema or both for more than 6 weeks. Although the symptoms of CU are thought to be caused, at least in part, by auto‐allergic mechanisms mediated by formation of immunoglobulin E (IgE), for example, against thyroid peroxidase or IL‐24,^1^ sensitization and/or allergy to external triggering factors are possibly not causative in CU. Still, allergic diseases such as asthma and hay fever,^2^ and sensitization to aeroallergens,^3^ are more prevalent in patients with CU, and the exact implications of this for patients with CU are not completely understood.

The oligosaccharide α‐gal (galactose‐α‐1,3‐galactose) is commonly expressed on non‐primate mammalian proteins and is capable of inducing delayed anaphylaxis, angioedema and urticaria after ingestion of red meat in patients with IgE specific for α‐gal.^4^, ^5^, ^6^ Little is known about α‐gal sensitization in patients with CU. Therefore, we examined two consecutive cohorts of newly referred patients with CU from: (1) a primary care allergology practice (*n* = 373) and (2) a tertiary hospital dermatology department (*n* = 283) plus a control group with allergic disease, but not CU, from the allergology practice (*n* = 77) for the prevalence, predictors and relevance of α‐gal sensitization. Patients were interviewed and screened for α‐gal sensitization (serum specific‐IgE ≥0.35 KU/L) using the ImmunoCAP™ (ThermoFisher).

In total, 21 (5.6%) and 11 (3.9%) of CU patients from private practice and hospital, respectively, were α‐gal sensitized, that is, they had specific IgE ≥0.35 KU/L, (*p* = 0.362 for difference between private practice and hospital). Based on, respectively, a class 2 (IgE ≥0.70 KU/L), class 3 (IgE ≥3.50 KU/L) and class 4 (IgE ≥17.50 KU/L) positivity‐criterion, the prevalence of α‐gal sensitization was 4.8%, 2.4% and 1.9% in private practice, and 2.8%, 1.4% and 0.4% in hospital. In total, 8 patients (38.1% of sensitized patients, and 2.1% of all CU patients) from private practice, and 2 patients (18.2% of sensitized patients, and 0.7% of all CU patients) from hospital, had relevant α‐gal allergy, respectively, judged on whether they had experienced systemic anaphylaxis, urticaria and/or angioedema up to 12 h after consumption of red meat. Levels of α‐gal‐IgE were numerically higher, but not statistically significantly different, in patients with relevant allergy compared to non‐allergic sensitized patients (median 36.95 vs. 1.81 KU/L, *p* = 0.108 in private practice; and 26.85 vs. 1.47 KU/L, *p* = 0.111 in hospital) and the fraction of relevant α‐gal sensitized patients was not higher in patients with higher IgE positivity classes. Among control patients from private practice the prevalence of α‐gal sensitization was 5.2% (*n* = 4) and none of these were relevant, *p* = 0.879 for difference from CU patients.

The following factors were the most evident discriminators between α‐gal sensitized and non‐sensitized patients from, respectively, private practice: male sex (47.6 vs. 24.7%), *p* = 0.020, obesity (33.3 vs. 23.6%), *p* = 0.302, and frequency of angioedema (61.9 vs. 51.4%), *p* = 0.350; and from hospital: male sex (72.7 vs. 27.9%), *p* = 0.003, and high total IgE (median 168 vs. 70.5 KU/L), *p* = 0.022 (Table 1).

**TABLE 1:** Characteristics of chronic urticaria and non‐urticaria patients from private allergology practice and tertiary dermatology hospital department according to α‐gal sensitization status

	Private allergology practice, *n* = 450				Dermatology hospital department, *n* = 283	
	Chronic urticaria, *n* = 373		Non‐urticaria, *n* = 77		Chronic urticaria, *n* = 283	
	α‐gal sensitized	α‐gal non‐sensitized	α‐gal sensitized	α‐gal non‐sensitized	α‐gal sensitized	α‐gal non‐sensitized
	*n* = 21 (5.6%)	*n* = 352 (94.4%)	*n* = 4 (5.2%)	*n* = 73 (96.1%)	*n* = 11 (3.9%)	*n* = 272 (96.1%)
Sex, *n* (%)						
Female	11 (52.4)	265 (75.3)	2 (50.0)	40 (54.8)	3 (27.3)	196 (72.1)
Male	10 (47.6)	87 (24.7)	2 (50.0)	33 (45.2)	8 (72.7)	76 (27.9)
Age (years), mean (SD)	54.0 (16.1)	45 (17.8)	68.2 (16.8)	48.1 (17.0)	33.8 (16.2)	38.2 (17.1)
Obesity (BMI ≥30.0 kg/m^2^), *n* (%)	7 (33.3)	83 (23.6)	0 (0)	18 (24.7)	N/A	N/A
Ever smoking, *n* (%)	9 (42.9)	160 (45.5)	2 (50.0)	29 (39.7)	5 (45.5)	111 (40.8)
Family history of CU, *n* (%)	N/A	N/A	N/A	N/A	2 (18.2)	51 (18.8)
Urticaria subtype, *n* (%)	N/A	N/A	N/A	N/A		
CSU					7 (63.6)	165 (60.7)
CINDU					2 (18.2)	46 (16.9)
CSU + CINDU					2 (18.2)	61 (22.4)
Age at onset, mean (SD)	46.9 (19.8)	N/A	N/A	N/A	29.9 (16.2)	33.1 (17.3)
Angioedema, *n* (%)	13 (61.9)	181 (51.4)	0 (0)	0 (0)	5 (45.5)	119 (43.8)
PRO‐score, mean (SD)	N/A	N/A	N/A	N/A		
UAS7					24.3 (16.6)	21.4 (14.1)
UCT					4.3 (3.9)	6.0 (4.2)
DLQI					8.7 (5.3)	8.8 (6.7)
VAS					6.3 (3.5)	6.0 (2.8)
Laboratory tests, median (min‐max)		N/A		N/A		
Eosinophils (E9/L)	0.20 (0.07–8.13)		0.32 (0.17–0.72)		0.13 (0.06–0.33)	0.14 (0.00–0.96)
Basophils (E9/L)	0.04 (0.00–0.10)		0.06 (0.03–0.07)		0.04 (0.01–0.06)	0.04 (0.00–0.17)
Neutrophils (E9/L)	3.90 (2.50–6.50)		3.77 (3.50–5.00)		2.69 (2.00–7.51)	3.70 (1.45–12.6)
CRP (mg/L)	1 (1.00–23.00)		1 (1.00–1.00)		1.00 (1.00–11.00)	1.00 (1.00–60.00)
Total IgE (KU/L)	233 (28–4.840)		103 (103–103)		168 (15–1.280)	70.50 (1.00–12.800)
TSH (E‐3 IU/L)	1.73 (0.75–3.42)		1.15 (1.08–1.21)		1.19 (0.31–4.01)	1.42 (0.01–8.16)
Positive BHRA, *n* (%)	N/A		N/A		1 (9.1)	30 (11.0)
Clinically relevant, *n* (%)		N/A		N/A		N/A
Yes	8 (38.1)		0		2 (18.2)	
No	6 (28.6)		3 (75.0)		7 (63.6)	
Information unavailable	7 (33.3)		1 (25.0)		2 (18.2)	

*Note*: Numbers are calculated from available data. Categorical variables are number (percentage) and continuous variables are mean (standard deviation, SD). Criterion for α‐gal sensitization is serum specific‐IgE >0.34 KU/L.

Abbreviations: BHRA, basophil histamine‐release assay; BMI, body mass index; CINDU, chronic inducible urticaria; CRP, C‐reactive protein; CSU, chronic spontaneous urticaria; CU, chronic urticaria; DLQI, dermatology life quality index; TSH, thyroid stimulating hormone; UAS7, urticaria activity score over 7 days; UCT, urticaria control test; VAS, visual analogue scale.

Among α‐gal sensitized CU patients from hospital with available data on specific IgE against meat (*n* = 8), 4 (50%) were also sensitized to beef, 4 (50%) were also sensitized pork, and 3 (37.5%) were also sensitized to lamb, whereas 4 (50%) were sensitized to at least one of these. Furthermore, 6 patients (54.5%) had a history of tick bite but none had ever been diagnosed or suspected infected with borrelia, and none had elevated IgG against borrelia.

Maurer et al.^7^ reported a prevalence of α‐gal sensitization of 2.4% in 83 patients with CU and no association between ingestion of red meat and development of subsequent delayed urticaria symptoms. In contrast, Pollack et al.^8^ found that 9 (60%) of 15 α‐gal sensitized CU patients had remission of urticaria symptoms due to avoidance of red meat or other mammalian‐derived food products. We have previously found that the prevalence of α‐gal sensitization (specific IgE ≥0.35 KU/L) in the general population from Copenhagen was 1.8%, and with higher age, cat ownership, positive skin prick test to common aeroallergens, and previous history of tick bite as statistically significant predictors in multivariable analysis for α‐gal positivity.^9^ Further, male sex was also a statistically significant predictor when the threshold for α‐gal positivity was lowered to IgE ≥0.10 KU/L.

In conclusion, we found that the prevalence of α‐gal sensitization among CU patients in private allergology practice and tertiary hospital dermatology department was low; 5.6% and 3.9%, respectively, with only few patients experiencing clinically relevant sensitization with allergic symptoms upon ingestion of red meat, respectively. Male sex and high total IgE levels were significant predictors of α‐gal sensitization. Particularly, the number of CU patients needed to screen to detect one case of clinically relevant α‐gal allergy was 46 (373/8) and 141 (283/2) in private practice and hospital, respectively. These results suggest that routine screening for α‐gal‐sensitization in patients with CU is relatively resource‐demanding and that only a small subgroup of patients, particularly from primary care, with certain characteristics, may constitute a relevant population for aimed testing. A potential role of known allergy to external triggers as a driver of auto‐allergy in CU needs further elucidation.

## AUTHOR CONTRIBUTIONS

Helena Swee‐Lin Trige Pedersen drafted the manuscript, made substantial contributions to the design, the acquisition, analysis and interpretation of data, revised the manuscript for intellectual content, approved the final version, and is accountable for all aspects of the work. Jennifer Astrup Sørensen, Flemming Madsen, Allan Linneberg, Katja Biering Leth‐Møller and Christian Vestergaard made substantial contributions to the design, the acquisition, analysis and interpretation of data, revised the manuscript for intellectual content, approved the final version, and is accountable for all aspects of the work. Simon Francis Thomsen initiated the work, made substantial contributions to the design, the acquisition, analysis and interpretation of data, revised the manuscript for intellectual content, approved the final version, and is accountable for all aspects of the work.

## CONFLICT OF INTEREST

The authors declare that there is no conflict of interest that could be perceived as prejudicing the impartiality of the research reported.

## FUNDING INFORMATION

Independent Research Fund Denmark, Grant/Award Number: 2034‐00031B.

## References

[1] Kolkhir P , Muñoz M , Asero R , et al. Autoimmune chronic spontaneous urticaria. J Allergy Clin Immunol. 2022;149(6):1819‐1831. 10.1016/j.jaci.2022.04.010 35667749

[2] Ghazanfar MN , Kibsgaard L , Thomsen SF , Vestergaard C . Risk of comorbidities in patients diagnosed with chronic urticaria: a nationwide registry‐study. World Allergy Organ J. 2020;13(1):100097. 10.1016/j.waojou.2019.100097 32021661 PMC6994395

[3] Wong MM , Keith PK . Presence of positive skin prick tests to inhalant allergens and markers of T2 inflammation in subjects with chronic spontaneous urticaria (CSU): a systematic literature review. Allergy Asthma Clin Immunol. 2020;16(1):72. 10.1186/s13223-020-00461-x 32944029 PMC7491258

[4] Commins SP , James HR , Kelly LA , et al. The relevance of tick bites to the production of IgE antibodies to the mammalian oligosaccharide galactose‐α‐1, 3‐galactose. J Allergy Clin Immunol. 2011;127(5):1286‐1293.e6. 10.1016/j.jaci.2011.02.019 21453959 PMC3085643

[5] Commins SP , Satinover SM , Hosen J , et al. Delayed anaphylaxis, angioedema, or urticaria after consumption of red meat in patients with IgE antibodies specific for galactose‐alpha‐1, 3‐galactose. J Allergy Clin Immunol. 2009;123(2):426‐433. 10.1016/j.jaci.2008.10.052 19070355 PMC3324851

[6] Chung CH , Mirakhur B , Chan E , et al. Cetuximab‐induced anaphylaxis and IgE specific for galactose‐alpha‐1, 3‐galactose. N Engl J Med. 2008;358(11):1109‐1117. 10.1056/nejmoa074943 18337601 PMC2361129

[7] Maurer M , Church MK , Metz M , Starkhammar M , Hamsten C , van Hage M . Galactose‐α‐1, 3‐galactose allergy is not a hitherto unrecognized cause of chronic spontaneous urticaria. Int Arch Allergy Immunol. 2015;167(4):250‐252. 10.1159/000440653 26426899

[8] Pollack K , Zlotoff BJ , Borish LC , Commins SP , Platts‐Mills TAE , Wilson JM . α‐Gal syndrome vs chronic urticaria. JAMA Dermatol. 2019;155(1):115‐116. 10.1001/jamadermatol.2018.3970 30476954 PMC6439572

[9] Gonzalez‐Quintela A , Dam Laursen AS , Vidal C , Skaaby T , Gude F , Linneberg A . IgE antibodies to alpha‐gal in the general adult population: relationship with tick bites, atopy, and cat ownership. Clin Exp Allergy. 2014;44(8):1061‐1068. 10.1111/cea.12326 24750173

